# The Role of *TP53* Mutations in *EGFR*-Mutated Non-Small-Cell Lung Cancer: Clinical Significance and Implications for Therapy

**DOI:** 10.3390/cancers14051143

**Published:** 2022-02-23

**Authors:** Matteo Canale, Kalliopi Andrikou, Ilaria Priano, Paola Cravero, Luigi Pasini, Milena Urbini, Angelo Delmonte, Lucio Crinò, Giuseppe Bronte, Paola Ulivi

**Affiliations:** 1 Biosciences Laboratory, IRCCS Istituto Romagnolo per lo Studio dei Tumori (IRST) “Dino Amadori”, 47014 Meldola, Italy; matteo.canale@irst.emr.it (M.C.); milena.urbini@irst.emr.it (M.U.); paola.ulivi@irst.emr.it (P.U.); 2 Department of Medical Oncology, IRCCS Istituto Romagnolo per lo Studio dei Tumori (IRST) “Dino Amadori”, 47014 Meldola, Italy; kalliopi.andrikou@irst.emr.it (K.A.); ilaria.priano@irst.emr.it (I.P.); angelo.delmonte@irst.emr.it (A.D.); lucio.crino@irst.emr.it (L.C.); giuseppe.bronte@irst.emr.it (G.B.)

**Keywords:** Non-Small-Cell Lung Cancer (NSCLC), Epidermal Growth Factor Receptor (*EGFR*), Tumor protein 53 (*TP53*), targeted therapy, resistance mechanisms

## Abstract

**Simple Summary:**

Non-Small-Cell Lung Cancer (NSCLC) is the primary cause of cancer-related death worldwide. Patients carrying Epidermal Growth Factor Receptor (*EGFR*) mutations usually benefit from targeted therapy treatment. Nonetheless, primary or acquired resistance mechanisms lead to treatment discontinuation and disease progression. Tumor protein 53 (*TP53*) mutations are the most common mutations in NSCLC, and several reports highlighted a role for these mutations in influencing prognosis and responsiveness to *EGFR* targeted therapy. In this review, we discuss the emerging data about the role of *TP53* in predicting *EGFR* mutated NSCLC patients’ prognosis and responsiveness to targeted therapy.

**Abstract:**

Non-Small-Cell Lung Cancer (NSCLC) is the primary cause of cancer-related death worldwide. Oncogene-addicted patients usually benefit from targeted therapy, but primary and acquired resistance mechanisms inevitably occur. Tumor protein 53 (*TP53*) gene is the most frequently mutated gene in cancer, including NSCLC. *TP53* mutations are able to induce carcinogenesis, tumor development and resistance to therapy, influencing patient prognosis and responsiveness to therapy. *TP53* mutants present in different forms, suggesting that different gene alterations confer specific acquired protein functions. In recent years, many associations between different *TP53* mutations and responses to Epidermal Growth Factor Receptor (*EGFR*) targeted therapy in NSCLC patients have been found. In this review, we discuss the current landscape concerning the role of *TP53* mutants to guide primary and acquired resistance to Tyrosine-Kinase Inhibitors (TKIs) *EGFR*-directed, investigating the possible mechanisms of *TP53* mutants within the cellular compartments. We also discuss the role of the *TP53* mutations in predicting the response to targeted therapy with EGFR-TKIs, as a possible biomarker to guide patient stratification for treatment.

## 1. Introduction

Lung cancer (LC) is the main cause of cancer-related death worldwide [1]. Non-Small Cell Lung Cancer (NSCLC), the most common LC histology, is a heterogeneous malignancy comprising molecular subtypes for which targeted agents are available in clinical practice [2]. Epidermal Growth Factor Receptor (*EGFR*) is the most common altered targetable gene in NSCLC, and its mutations (mainly exon 19 deletions and exon 21 L8585R mutations) represent the predictive biomarker for first-, second- and third-generation *EGFR*-Tyrosine Kinase Inhibitors (TKIs). Nonetheless, resistance to *EGFR* targeted therapy inevitably occurs, and molecular mechanisms at the basis of primary and acquired resistance to TKIs still have to be elucidated [3].

Tumor protein 53 (*TP53*) gene encodes for p53 protein, a transcription factor recognized as a master regulator of a wide range of cellular processes such as proliferation, differentiation, apoptosis, metabolism and DNA repair [4]. Gene alterations affecting *TP53* are the most commons across all cancers, and are involved in cancer onset, development, progression and response to therapies, thus also affecting the patient’s prognosis [4].

To date, several *TP53* mutations have been highlighted, with relative peculiar protein associated functions; more than 70% of these alterations are represented by missense mutations along the DNA-binding domain (DBD), resulting in different consequences at a cellular, organismal and clinical level [4]. Gene alterations affecting *TP53* are proved to be a strong prognostic factor for NSCLC [5], and recent reports indicate a role for these mutations in predicting *EGFR*-mutated NSCLC patients responsiveness to TKIs. In this review, we focus on the relation between *TP53* mutations and *EGFR*-mutated NSCLC subtype, discussing the achieved results on the role of such mutations in predicting responsiveness to TKIs. We also explore possible cellular mechanisms that *TP53* mutants activate to guide the resistance to therapy and discuss the emerging data on the role of these gene mutations for a possible patient stratification for EGFR-mutated patients. Moreover, we discuss the classification systems proposed to date, as it has been demonstrated that different mutations confer different characteristics to the cancer cell.

## 2. *EGFR*-Mutated NSCLC

LC is the main cause of cancer-related mortality worldwide (18.4%), while NSCLC accounts for 80–85% of lung cancers [1]. In patients affected by metastatic NSCLC, clinical guidelines recommend the testing of activating mutations, the majority of which are linked with an activation of *EGFR* that occurs in 10–20% of Caucasian and 50% of Asian patients [1,6].

The *EGFR* gene is located in the short arm of chromosome 7 (7p11.2) [7]. *EGFR* belongs to the HER/ErbB2 family, a group of receptor tyrosine kinases that include epidermal receptor tyrosine kinases 1 (*EGFR*, ERBB1), HER2/ERBB2, HER3/ERBB3 and HER4/ERBB4 [8]. These receptors share a similar structure and are composed of three regions: the intracellular, the extracellular, and the transmembrane regions [9]. When the receptor is activated by the ligand, it dimerizes and autophosphorylates the tyrosine residues in the cytoplasmic domain. This step consequently allows the triggering of the intracellular signaling and gene transcription process [9]. The intracellular signaling cascade is mediated through the following pathways: RAS/RAF/MEK/MAPK, PI3K-AKT, JAK/STAT, Src kinase, the Endocytic pathway [10]. Downstream *EGFR* signaling influences gene expression, apoptosis inhibition, proliferation, angiogenesis, cell motility, and metastasis [6,7,9,11].

The *EGFR* activity can be dysregulated by several activating mutations that occur within the exons from 18 to 21 (encoding the kinase domain) [8]. Exon 19 deletions (ex19Del) of amino acids 747–750 account for 45% of all *EGFR* mutations, while exon 21 mutations account for 40–45% and are characterized by the substitution of leucine for arginine (L858R) [11]. The remaining 10% are uncommon mutations affecting exons 18 and 20. Among these, the most frequent (4–8%) is the exon 20 insertion, which is also associated with resistance to the three generations of *EGFR* inhibitors [6]. Ex19Del and L858R are responsible for a constitutional activation of the receptor: ex19Del shortens the activation loop and prevents the rotation of the alpha helix causing a destabilization of the inactive conformation. L858R causes the interaction between the N-lobe and the C-lobe in the inactive conformation. L858R causes steric hindrance and leads to a constitutive active conformation [6].

### 2.1. EGFR+ Clinical Management: Clinical Trials Demonstrating First-, Second- and Third-Generation TKIs Efficacy

#### 2.1.1. First-Generation of *EGFR* TKIs

Gefitinib and erlotinib are first-generation reversible *EGFR* inhibitors. The activity of gefitinib and erlotinib were initially evaluated in unselected NSCLC patients with poor results [12]. Despite these disappointing results, a retrospective analysis of responders to these treatments allowed the researchers to identify *EGFR* mutations as predictive biomarkers for responsiveness to *EGFR* TKIs.

The IPASS trial was the first randomized phase III trial evaluating the efficacy of gefitinib versus chemotherapy (carboplatin/docetaxel) in 1217 treatment-naïve *EGFR*-mutated NSCLC patients. This study met its progression-free survival (PFS) primary endpoint and objective response rate (ORR) endpoint [13]. Indeed, the PFS (9.5 months vs. 6.3 months; HR 0.48, *p* < 0.001) and ORR (71.2% vs. 47.3%) in the gefitinib arm was superior to those of the chemotherapy group. It is of note that this study confirmed that *EGFR* mutations are the strongest predictive biomarker for response to front-line gefitinib. The phase III NEJ002 study confirmed the superiority in terms of PFS gefitinib compared to carboplatin and paclitaxel (10.8 months vs. 5.4 months; HR 0.3; *p* < 0.001) [14]. Similarly, in the phase III WJT0G3405 trial, PFS was higher in the gefitinib arm than in the carboplatin/docetaxel arm (9.2 vs. 6.3 months, HR 0.489, *p* < 0.0001) [15]. Moreover, two phase III trials compared erlotinib to chemotherapy confirming the superiority of this *EGFR* TKI in terms of PFS [16,17]. As shown in Table 1, a total of six large phase III trials showed a strong benefit of *EGFR* TKIs versus chemotherapy as a first-line treatment in terms of PFS and ORR in patients with *EGFR*-mutated NSCLC [13,14,15,16,17,18,19]. However, these studies did not demonstrate a significant benefit in Overall Survival (OS), probably due to the high cross-over rate between the Gefitinib or Erlotinib arm and the chemotherapy arm [20].

Based on these results, gefitinib was approved by the FDA (US Food and Drug Administration) in 2015 and erlotinib in 2016 for the treatment of patients with metastatic NSCLC whose tumors have *EGFR* exon 19 deletions or exon 21 (L858R) substitution mutations.

The efficacy of another first-generation *EGFR*-TKI, namely icotinib, has been evaluated in a phase III CONVINCE trial. In this first-line study, icotinib showed a higher PFS in comparison with the chemotherapy arm (11.2 months vs. 7.9 months; HR 0.61; *p* = 0.06) [21]. Therefore, based on CONVINCE results, icotinib was approved by the China Food and Drug Administration (CFDA) in June 2011 as a first-line treatment, enriching the *EGFR*-mutated NSCLC therapeutic armamentarium.

The evolution of the *EGFR* TKIs in the therapeutic landscape entailed the need to define a better first-line strategy treatment in this patient setting. Therefore, the phase III CTONG 0901 trial was conducted to compare the efficacy and safety of gefitinib with that of erlotinib in patients with metastatic NSCLC characterized by *EGFR* exon 19 or 21 mutations [28]. The results of this comparison demonstrated that the PFS and OS of the erlotinib and gefitinib arms were 13.2 months vs. 11.1 months (HR 0.80; *p* = 0.108) and 22.4 vs. 20.7 months (HR 0.98; *p* = 0.902) respectively. Even though this study did not meet its primary endpoint, the subgroup analyses demonstrated that patients with *EGFR* exon 19 mutations had a significantly higher RR (62.2% vs. 43.5%, *p* = 0.003) and superior median OS (22.9 vs. 17.8 months, *p* = 0.022) than those with exon 21 mutations treated with erlotinib or gefitinib.

The ICOGEN trial is another randomized, double-blind, phase III trial that was conducted to evaluate the safety and efficacy of icotinib and gefitinib in advanced NSCLC patients previously treated with chemotherapy [29]. In this study, the PFS (7.8 months for icotinib vs. 5.3 months for gefitinib, *p* = 0.32, and OS 20.9 vs. 20.2; *p* = 0.76) were similar between the two arms, but the toxicity of the icotinib arm was lower than that of the gefitinib arm (60.5% vs. 70.4%). Thus, compared with the results of these two head-to-head studies, the three first-generation *EGFR*-TKIs do not seem to be significantly different in terms of the PFS and safety profile.

#### 2.1.2. First-Generation *EGFR* TKIs Combined with Other Agents

Based on the evidence of a link between *EGFR* stimulation and increased angiogenesis, first-generation *EGFR* TKIs were tested in combination with anti-angiogenic compounds. An anti-vascular endothelial growth factor (anti-VEGF) +  erlotinib combination has been investigated in patients with untreated, advanced, *EGFR* mutated NSCLC in the phase II JO25567 trial, which evaluated the efficacy of erlotinib and bevacizumab compared with erlotinib alone in patients with EGFR mutation-positive NSCLC. The PFS benefit was more consistent for the erlotinib + bevacizumab arm than in the erlotinib arm (median, 16.0 vs. 9.7 months; HR, 0.54; 95% CI, 0.36–0.79; *p* = 0.0015), leading to EMA approval for this combination [30].

A phase III trial evaluated the efficacy of the addition of ramucirumab (anti-VEGF receptor inhibitor) to erlotinib, finding a significant benefit in PFS (19.4 vs. 12.4 months, HR: 0.59, *p* < 0.0001) [31]. Based on these results, despite the fact that the data of OS are immature, this combination has been approved by the FDA (US Food and Drug Administration) for the first-line treatment of patients with metastatic NSCLC harboring *EGFR* exon 19 deletions or exon 21 mutations. Interestingly, a biomarker analysis from the RELAY trial highlighted that p53 and EGFR co-mutations were associated with prolonged PFS in the experimental arm, both in exon 19 and 21 mutated patients [32]. The addition of chemotherapy to first-generation TKIs was also investigated. A recent meta-analysis of randomized controlled trials that compared *EGFR*-TKI monotherapy with the combination of *EGFR*-TKI and chemotherapy showed that there was a benefit in terms of ORR, PFS and OS in favor of the combination arm, with an acceptable toxicity profile. These data are of interest and the combination of chemotherapy with *EGFR* TKIs could be a potential first-line treatment in selective patients [22].

#### 2.1.3. Second-Generation of *EGFR* TKIs

Afatinib and dacomitinib are second-generation irreversible *EGFR* TKIs with a similar structure with that of gefitinib or erlotinib but with a side chain that binds covalently to cysteine-797 of *EGFR* with a subsequent irreversible *EGFR* inhibition [33,34].

Afatinib was evaluated as a first-line treatment in comparison with cisplatin and pemetrexed in the phase III LUX-Lung 3 trial, demonstrating superiority in terms of PFS compared to chemotherapy (11.1 months vs. 6.9 months; HR 0.58; *p* = 0.001). In this trial, only patients with common *EGFR* mutations (exon 19 deletions and L858R) were considered, with a reported increase in PFS of 13.6 months for the experimental arm vs. 6.9 months for the chemotherapy arm (HR = 0.47; 95% CI, 0.34–0.65; *p* = 0.001). Interestingly, the PFS result was superior in patients with tumours harboring an exon 19 deletion with respect to the L858R mutation [35]. On the basis of this clinical trial result, afatinib was approved as a treatment for treatment-naïve patients affected by advanced *EGFR*-mutated NSCLC. In the LUX-Lung 6 trial, patients were randomized to receive afatinib or gemcitabine/cisplatin chemotherapy. The final results of this study showed a statistically significant benefit in terms of the PFS for the afatinib arm (11.0 vs. 5.6 months; HR 0.28; *p* < 0.0001) [36]. Despite the PFS benefit for afatinib in these two trials, no difference in terms of survival benefit in the overall population was reported. However, in a prespecific EGFR exon 19 subgroup analysis, afatinib demonstrated a significant improvement in OS compared with the chemotherapy group, in both trials. On the other hand, in the LUX-Lung 7 study, a head-to-head comparison between afatinib and gefitinib, no significant differences in terms of PFS were found (11.0 months for afatinib vs. 10.9 months for gefitinib; HR 0.73; *p* = 0.017) [24].

Another second-generation *EGFR* inhibitor, dacomitinib, was evaluated in the phase III ARCHER-1050 trial [37]. A total of 452 patients with *EGFR*-mutant NSCLC were randomized to receive as a first-line treatment dacomitinib 45 mg/day or gefitinib 250 mg/day. The results of this trial demonstrated that the PFS of the dacomitinib was statistically longer compared with the gefitinib arm (14.7 months vs. 9.2 months HR: 0.59; 95% CI: 0.47–0.74). In responders, the duration of response was longer in the dacomitinib arm vs. the gefitinib arm in both *EGFR* mutation subgroups. However, a dose reduction of dacomitinib was still needed in 66.5% of the trial patients due to a more significant toxicity profile of dacomitinib. Moreover, mOS was significantly longer in the dacomitinib arm than in the gefitinib arm (34.1 months vs. 26.8 months, respectively; HR 0.76; 95% CI, 0.582 to 0.993; *p* = 0.0438). In the updated OS analysis, a significant improvement in survival was confirmed for the dacomitinib group in patients with exon 21 (L858R) substitution mutations (32.5 months vs. 23.2 months; HR 0.67; 95% CI: 0.47–0.94; *p* = 0.02); whereas, there was no survival benefit in patients with the exon 19 deletion mutation (36.7 months for dacomitinib vs. 30.8 months for gefitinib, HR 0.85; *p* = 0.30) [23].

#### 2.1.4. Third-Generation of *EGFR* TKIs

One of the most common mechanisms of acquired resistance during the treatment with first- and second-generation *EGFR* TKIs is represented by the mutation of T790M (*EGFR* exon 20). Therefore, clinical development was focused on second-line treatments with the subsequent development of third-generation *EGFR* TKIs. Osimertinib is an oral, third-generation, irreversible *EGFR* TKI designed to have activity against T790M mutations as well as activity in the central nervous system [27]. The phase III AURA3 trial evaluated the effects of osimertinib or cisplatin/pemetrexed in randomized *EGFR*-positive patients with T790M resistance [38]. This study demonstrated a significant improvement in PFS, with a median of 10.1 months in the osimertinib arm compared to 4.4 months with chemotherapy (*p* < 0.001). Furthermore, osimertinib was demonstrated to be superior to chemotherapy in terms of ORR (71% vs. 31%) and with an intracranial ORR of 70%. Based on the results of this study, osimertinib was approved as a second-line treatment of *EGFR* T790M-mutated NSCLC patients.

A sequent phase III FLAURA study assessed the efficacy and safety of osimertinib in patients with previously untreated *EGFR* mutated advanced NSCLC compared with the standard first-generation *EGFR*-TKIs (gefitinib or erlotinib) [39]. The results of the FLAURA trial showed that osimertinib resulted in significantly longer PFS (18.9 months vs. 10.2 months; HR 0.46; *p* < 0.001) with a better safety profile with respect to standard *EGFR*-TKIs. Despite crossover by 31% of the patients in the control arm, osimertinib confirmed its superiority in terms of OS with a median OS of 38.6 months (95.05% CI, 34.5–41.8) vs. 31.8 months (95.05% CI, 26.6, 36.0) for standard *EGFR* TKI treatment, respectively (HR 0.799; 95.05% CI, 0.641–0.997; *p* = 0.0462). Based on these practice-changing results, osimertinib was approved as a first-line treatment for patients with metastatic NSCLC harboring *EGFR* exon 19 deletions or exon 21 L858R mutations.

### 2.2. EGFR TKIs for the Treatment of Uncommon EGFR Mutations

Approximately 10–15% of *EGFR*-mutated tumors present uncommon somatic *EGFR* mutations such as exon 18 nucleotide alterations, exon 19 in frame insertions, exon 20 alterations, and exon 21 mutation L861Q. The knowledge concerning the efficacy of *EGFR* TKIs in patients affected by NSCLC carrying uncommon *EGFR* mutations is limited to small clinical investigations or case reports. A post hoc analysis of the LUX-Lung 2, LUX-Lung 3, and LUX-Lung 6 trials demonstrated that patients with L861Q, G719X and S768I responded well to afatinib treatment, whereas afatinib did not show any efficacy in patients with an exon 20 insertion mutation [40,41]. Another retrospective study confirmed the minor efficacy of first-generation *EGFR* TKIs in patients with G718X and L861Q [42]. Encouraging results come from the clinical activity of amivantamab, a bispecific antibody directed against *EGFR* and *MET* tyrosine kinase receptors, and against tumors with *EGFR* exon 20ins mutations [43]. However, we have to wait for the conclusion of ongoing phase III studies to better define its role in this patient setting. Moreover, osimertinib efficacy was tested in a phase II trial of enrolled patients with uncommon EGFR mutations, demonstrating a 50% objective response rate and 8.2 median PFS [44].

## 3. Common Resistance Mechanisms to *EGFR*-TKIs

Despite the known and confirmed efficacy of *EGFR*-TKIs in the treatment of *EGFR* mutated NSCLC, about 5–25% of patients, who receive target therapies, show no benefits from this treatment [45]. The cause of this resistance is the presence of mechanisms that, generally, could be grouped into on-target *EGFR*-dependent and off-target *EGFR*-independent. Moreover, primary, or secondary resistance mechanisms could be observed, depending on whether they occurred from the beginning of the treatment with *EGFR*-TKI or after an initial period of response or stability.

### 3.1. On-Target Mechanisms of Resistance

On-target mechanisms of resistance occur mostly with the use of first- and second-generation *EGFR*-TKIs at about 50%, compared to 10–15% for third-generation TKI used as a first-line and 20% as a second-line [3]. One of the most known on-target mutations is T790M, characterized by a threonine substituted with a methionine in the 720 position of exon 20. T790M mostly develops as a resistance mechanism to first- and second-generation *EGFR*-TKIs used as a first-line treatment, occurring in about 49–63% of patients [46,47,48]. Finding this mutation as a diagnosis of a primary mechanism of resistance is rare. In this case, a possible germinal origin of the mutation should be considered [46]. Other reported rare alterations conferring resistance to first- and second-generation *EGFR*-TKIs are the missense mutations D761Y, L747S and T854A, while *EGFR* amplification occurs in 8–10% of cases.

On the other hand, some on-target missense mutations conferring resistance to osimertinib have been identified. Exon 20 C797S is able to avoid the binding of osimertinib to *EGFR*, as well as other third-generation TKIs (e.g., rociletinib, narzatinib, olmutinib) [49,50]. This mutation is the most common tertiary mechanism of resistance and accounts for about 10–26% of cases resistant to osimertinib when used as a second-line treatment; although, when it is used as a first-line therapy, its prevalence is 7% lower, though it remains the second most frequent event behind *MET* amplification [38,51]. The same effect occurs with mutations affecting L792. On the other hand, it was demonstrated that this resistance mutation to third-generation TKIs is sensible to first-generation gefitinib in vitro [46,50,51]. Another mutation interfering with the binding of osimertinib is the substitution in the 718 residue (L718Q, L718V), placed in the adenosine triphosphate (ATP) binding site. Usually, these mutations confer independent resistance to osimertinib, not coexisting with the C797 mutation [52,53]. Anecdotal and rare mutations interfering with osimertinib activity include C797G and mutations in the C796 residue (C796R, G796S, G796D) in exon 20, near C797 [49,52,54,55,56,57]. In the same protein domain, the G719A mutation has been reported in association with osimertinib resistance. Similarly, other exon 20 mutations (G724S and SV768IL) induce resistance to third-generation TKIs [50,53,56,57]. Finally, the amplification of *EGFR* and a deletion in exon 19 have been described as additional mechanisms of resistance to osimertinib in the II line setting [58,59]. Lazertinib, a third-generation TKI targeting activating and T790M *EGFR* mutations, showed important clinical activity in EGFR-mutated NSCLC patients as a second-line treatment after progression to a first- or second-generation TKI [60] and its efficacy, in combination with amivantamab after progression to osimertinib treatment, was demonstrated [61].

### 3.2. Off-Target Mechanisms of Resistance

Off-target resistance mechanisms induced by TKI treatment include alterations affecting pathways that bypass signaling activation, in an *EGFR*-independent manner. As *TP53* mutations involved in the process of histologic transformation to SCLC are a resistance mechanism to *EGFR*-TKIs, this issue is discussed later.

#### 3.2.1. *MET* Amplification

*MET* amplification is one of the most frequent mechanisms of resistance to all generations of *EGFR*-TKIs, used as both a first- and second-line of treatment.

It was reported in 5–22% of cases treated with first- and second-generation TKIs, mostly in association with an *EGFR* exon 19 deletion, while it is the most common cause of resistance to osimertinib used in II line [62,63,64]. This data has been observed in the AURA3 trial, where about 19% of the cfDNA samples at the progression showed MET amplification, though the percentage was lower in tumor tissue [53,62,64]. In this setting, MET amplification occurs with or without the loss of T790M, and in 7% of cases could be present with a C797S mutation [63,65].

#### 3.2.2. HER2

The tyrosine kinase receptor Erb2, encoded by HER2, stimulates the activation of the MAPK and PI3K pathways, mediating resistance to *EGFR*-TKIs. HER2 amplification has been described in 12% of tumor samples of patients treated with first-generation TKI [66], in 5% of cases treated with osimertinib in II line, and in 2% of cases treated in I line [65,66,67]. This last percentage has been found when the cfDNA was analyzed, and no HER2 amplification was detected in the tumor tissue.

#### 3.2.3. *KRAS*, BRAF and PI3K

*KRAS* mutation as a mechanism of resistance to *EGFR*-TKIs is very rare. G12S mutation has been described as resistant to osimertinib in II line; other *KRAS* mutations, such as G12D, G13D, Q61R and Q61K, have been found to confer resistance to osimertinib as well (reported in less than 1% of cases) [63,65,68,69,70]. In the FLAURA study, mutations of *KRAS*, like G12D and A146T, were described in the cfDNA of 3% of cases [51]; in the study conducted by Schoenfeld on tumor tissue, *KRAS* mutation G12A was recorded in one case (4%) [54]. Another mechanism of resistance observed is the BRAF V600F; after treatment with osimertinib in II line, it was observed in 3% of the cfDNA samples [65]. The same frequency was also reported in the cfDNA analyzed after progression with osimertinib used in I line [54]. At progression after osimertinib is used as a second-line treatment, the *PI3KCA* mutations E454K, E452K, R88Q, N345K, and E418K, have been identified as mechanisms of resistance in 4–11% of cases [62,64,68,70]; in another study, the percentage of these mutations noted on tumor tissue was 17% [71]. Moreover, *PI3KCA* amplification has been detected, using NGS, in the AURA3 study in 4% of the cases [65]. The main clinical trials investigating new therapeutic strategies for patients with progressive disease after treatment with Osimertinib are resumed in Table 2.

## 4. *TP53*: The Master Regulator of the Genome

The 53 KD protein p53, encoded by the Tumor protein P53 (*TP53*) gene is a master regulator of cell fate and a powerful antiproliferative transcription factor that controls the epigenetic program and dictates the expression of a plethora of target genes in response to multiple external stresses [72]. In a balanced homeostasis cell state, p53 is maintained at low cytoplasmic levels and kept mostly inactivated by the regulatory action of the E3 ubiquitin-protein ligase, MDM2; various cellular stimuli, including DNA damage and replication induced by oncogenic deregulation, releases MDM2-mediated degradation of p53 and promotes p53 activation by its phosphorylation [73].

The best understood function of p53 focuses on its DNA-binding ability to induce cell cycle arrest and promote apoptosis [74]. However, p53 also plays a pivotal role in controlling the overall integrity of the genome, as it is often addressed as the “guardian of the genome”. Upon DNA damage, multiple signaling pathways converge to activate p53, either promoting the repair of the damaged DNA sequence or blocking the DNA replication fork, and in doing so, the propagation of genomic instability and mutations [75]. In addition, p53 can limit chromosomal rearrangements by blocking centromere duplication and telomeric dysfunctions that lead to aberrant mitosis [76]. Mainly for this reason, the absence or inactivation of p53 permits cell survival and facilitates aneuploidy, which is a common step towards further accumulation of oncogenic abnormalities. In addition, p53 suppresses shattering genomic rearrangements such as chromothripsis that typically occur when cells have bypassed replicative senescence [77]. Although the extent of chromothripsis contribution to oncogenesis is still open to debate, this phenomenon is significantly more recurrent when *TP53* is deleted or mutated [78]. Another important way by which p53 contributes to maintaining genomic integrity is via the suppression of retrotransposon reactivation and mobilization that can lead to mutagenesis throughout the genome [79]. Specifically, p53 binding to long interspersed nuclear element (LINE) elements promotes LINE epigenetic silencing and might protect the cell from transposon-associated mutagenesis [80].

The control of genome integrity by p53 extends to multiple layers that are far beyond its well-known function as a transcription factor [72]. Several genome-wide studies have shown that p53 possesses repressive functions that directly depend on the DNA-binding capacity of p53 [81,82]. ChIP-Seq genome scanning revealed the presence of up to 10,000 possible positions in the genome that p53 potentially binds, which are widespread and do not always represent the binding preferences of p53 as a transcription factor [83]. Furthermore, many of the transcriptionally active promoters bound by p53 do not display direct p53-dependent regulation, suggesting the existence of different dynamics through which p53 might regulate the chromatin status and the overall stability of the genome [81]. For example, p53 can bind with high affinity to regions of chromatin in a closed status [84]. These findings may also raise the hypothesis that it is the nucleosomal structure rather than the DNA sequence affinity that dictates the genomic binding pattern of p53. A comparison between normal and cancer cells suggested that enrichment in CpG islands and hypomethylation of the DNA may drive p53 binding, which likely arise from the overall altered structure of chromatin during oncogenic transformation [85]. In a context of DNA damage, p53 can form a complex with the remodeling and splicing factor 1 (RSF1) forming a complex with the histone acetyltransferase p300 [86].

How the interplay between the transcription factor and the chromatin remodeling activity of p53, in a normal cellular context or upon the induction of DNA damage, affects the cell fate and is a question currently awaiting answers; it might have important implications when thinking about cancer therapy. In this concern, p53 inactivation that occurs in cancer may be unique in its ability to both favor genomic instability and sustain survival by downgrading p53 activity as a transcriptional repressor.

### TP53 Mutations in Human Cancer

The *TP53* gene has long been recognized as the most frequently mutated gene in human cancer [87]. It is, in fact, well accepted that various mutations in the *TP53* gene are the most common genetic lesions found in cancer cells, and mutational dysfunction of the p53 protein is a major contributor to cancer development, progression, metastasis, and resistance to therapy. Further, the presence of mutations that abrogate p53 functionality could also predispose a patient to resistance to cancer therapy [88]. Still, no effective medication that can block the oncogenic derangements derived from p53 inactivation has been approved for use in clinic. The inadequacy in restoring tumor suppressor activity of p53 mutants might also depend on the variety of effects that the different p53 mutations have on the cell [4]. Nevertheless, the precise characterization, in terms of functionality and pathological consequences, of the various p53 mutations is particularly relevant for their use as clinical biomarkers and the optimization of therapeutic options.

Most frequently, *TP53* mutations in cancer cells occur in one allele, while the other allele has been lost or deleted following major chromosomal rearrangements, leading to a loss of heterozygosity (LOH) that results in the expression of the sole mutated *TP53* allele [89]. However, a good fraction of tumors do not present with LOH for *TP53*, indicating that mutations of the gene might not be necessarily the primary driver of oncogenesis, but might occur at later stages and be just one among many critical pathological events that accumulate during a cancer cell’s life. Typical alterations of *TP53* include frameshifts (deletions and insertions), nonsense, silent, and missense mutations that may occur throughout the entire gene sequence [4]. Missense mutations are by far the most common alterations of *TP53* (>70%), and normally cluster in the DNA-binding domain (DBD, exons 5–8). Minor hotspot mutations may occur in other coding regions of the gene that are still associated with amino acid residues responsible for the interaction of the protein with DNA. Typically, all of these *TP53* mutations have been collectively considered equally for their ability to interfere with the tumor suppressor activity of p53, but there are actually profound differences in the classes of mutations, which may produce distinct outcomes [4].

The main function of p53 as a tumor suppressor is linked to its ability to induce cell death or to put the cell into a permanent senescent status. However, it is now quite established that several gain of function (GOF) mutations may happen in *TP53*, and sustains the notion that cancer cells may actually be addicted to mutated p53 [90]. A depletion of mutated p53 leads to cancer cell death, while ectopic expression of mutant p53 promotes survival via increased genomic instability, angiogenesis, and invasion [91,92,93]. Moreover, recent discoveries unveiled the capacity of p53, in certain contexts, to promote cell survival by also sustaining metabolism and maintaining the balance between glycolysis and oxidative stress, thus, limiting the production of reactive oxygen species [94]. Specifically, chances of p53 levels over time rather than its absolute levels are pivotal in driving cell fate and determining how the cell can respond to perturbations [95]. For example, during treatment with the chemotherapeutic agent Cisplatin, the mRNA stability of p53 target genes, which respond to p53 temporal regulation, is the main determinant in deciding whether the cell will survive to treatment [96]. This notion contributes to the question that multiple levels of p53 regulation may exist, and deciphering the complexity of p53 function relies upon the integration of the tumor suppressive activity of p53 and the understanding that deregulation of some elements of the p53 pathway might also provide the tumour with a survival advantage. Metabolic status, the overall mutational profile, and the epigenetic state of the cell are all determinants of how the tumor suppressive function of intact p53 might be restored during cancer treatment.

From a clinical perspective, targeting GOF mutations of p53 may have direct effects on the proliferation and survival of cancer cells that are addicted to p53. However, drugs that target the mutant form of p53, either to block GOF activity or to restore the tumor suppressive activity of p53, should have little interference on the wild-type p53. In particular, restoration of the wild-type function of p53 has been heavily pursued [97]. After almost four decades of studying, p53 is still considered undraggable, especially for the numerous off-target effects that many compounds, initially found as being able to target specifically p53 mutants, actually have. In particular, drugs that promote degradation of mutant p53, have adverse effects on many ubiquitous cellular pathways [98]. Similarly, use of non-selective anti-MDM2 inhibitors that should act in enhancing wild-type p53 activity have been revealed to be problematic due to many adverse side effects in patients [98].

This negative trend might, however, soon change, thanks to the availability of novel FDA-approved drugs, already being tested in clinical trials, that are more specific for individual p53 mutations that stratify with patient characteristics [4]. The evolving understanding of the specific functions of the different p53 mutant types might open the door, in the near future, to effective mutant p53 directed therapeutic strategies that will benefit over 50% of cancer patients, particularly those patients with *TP53* mutations.

## 5. *TP53* Mutations in NSCLC

As with the majority of cancers, *TP53* is the most common mutated gene in NSCLC, also [99,100,101] showing a predominant clonal expression [102]. Data analysis from The Cancer Genome Atlas (TCGA) database highlights that *TP53* mutations occur in the exons 4–8 of the gene in 44.8% of cases, confirming that DBD represents the hotspot of the protein. The authors showed that *TP53* mutations are able to affect the prognosis of NSCLC patients (OS 27 vs. 19 months, *p* < 0.001); moreover, different mutations result in different survival times, suggesting that different mutations play different roles at a molecular level [5]. Several studies identified that mutations affecting *TP53* are the most influencing prognostic factor, both in early and advanced NSCLC [103,104,105,106]. Moreover, it has been recently reported that activation of *TP53* is involved in the *EGFR-*signaling pathway and in the apoptosis process induced by platinum-based chemotherapy [107]. This evidence, together with the fact that *TP53* mutations are more frequent in *EGFR*-mutated patients, suggest that some of these oncogene-addicted tumors could possess an underlying biology and molecular mechanisms based on two main biomarkers to guide cancer progression [5,108]. On the other hand, in an attempt to find predictive biomarkers for a neo-adjuvant therapy for stage II–III *EGFR*-mutated NSCLC, exon 4/5 *TP53* missense mutations have been found to be a stratification factor for OS and treatment [109]; another study based on the IALT trial case series, found that *TP53* mutations play a role in predicting the efficacy of adjuvant platinum-based chemotherapy [110]. In the next paragraph, we discuss the emerging role of *TP53* in *EGFR*-mutated patients, both in terms of prognostic impact and resistance to targeted therapy.

### 5.1. TP53 Mutations in EGFR-Positive NSCLC: Clinical Significance

The role of *TP53* mutations in *EGFR*-mutated NSCLC has been widely investigated in recent years. One of the first attempts to establish the role of *TP53* mutations in this subset of patients was performed by Molina-Vila and colleagues, who explored the prognosis of 125 wild-type (wt) *EGFR* and 193 (training cohort) and 64 (validation cohort) mutated *EGFR* NSCLC patients. The authors categorized *TP53* mutations as disruptive and non-disruptive ones. Disruptive mutations were identified in stop codons along all the protein structures and non-conservative mutations within the DBD, while non-disruptive mutations were conservative alterations, as well as non-conservative mutations outside the DBD, apart from the stop codons. Their results showed that *TP53* non-disruptive mutations are an *EGFR*- and KRAS-independent prognostic factor (OS 13.3 months vs. 24.6 months, *p* < 0.001); they also showed that non-disruptive mutations are a prognostic but not a predictive factor in the subgroup of *EGFR*-mutated patients (median OS 17.8 months vs. 28.4 months in the training cohort, *p* = 0.004; median OS 18.1 months vs. 37.8 months in the validation cohort, *p* = 0.006), highlighting a slight trend in the PFS in a multivariate analysis of erlotinib-treated patients (PFS 11.0 vs. 15.0 months, *p* = 0.14) [111]. Similar results were recently achieved by Aggarwal and colleagues, who highlighted a worse OS for 114 *EGFR* and *TP53* co-mutated patients treated with first-line TKIs with respect to sole *EGFR* patients (median OS 33.3 months vs. 53.6 months; *p* = 0.021), with a trend when the data were adjusted for age, smoking status, and performance status [112]. A large cohort study showed a trend for *TP53* mutations in predicting a worse OS of *EGFR*-mutated patients receiving targeted therapy with respect to wt *TP53* patients (2.9 years vs. NR, *p* = 0.06); the trend became more evident when categorizing *TP53* mutations as disruptive and non-disruptive (*p* = 0.055), reaching statistical significance when pooling data from patients with targetable alterations in *EGFR*, *ALK* and *ROS1* genes (mOS 2.6 years vs. NR, *p* = 0.009) [113]. In the attempt to find predictive biomarkers for *EGFR*-mutated NSCLC patients, our group highlighted that mutations in *TP53* are able to influence responsiveness to first-line first- and second-generation TKIs in a case series of 136 *EGFR*-mutated NSCLC patients. In particular, *TP53* exon 8 mutations were able to identify a subset of patients with worse DCR with respect to wt exon 8 patients (41.7% vs. 87.3%, *p* < 0.001) PFS (4.2 vs. 16.8 months, *p* < 0.001) and OS (7.6 months vs. NR, *p* = 0.006), with respect to *TP53* wt and mutated *TP53* in other exon patients [114]. Later, we confirmed our results in an independent case series of *EGFR*-mutated patients (HR for PFS 3.16, 95% CI 1.59–6.28, *p* = 0.001) [115]. These results were confirmed in a larger case series of NSCLC patients, where *TP53* exon 8 mutations were able to predict the prognosis of *EGFR*-mutated patients, independently of the received treatment [116]. These results were not confirmed by the abovementioned study, which found that *TP53* in exon 8 were not predictors for PFS compared with mutations in other exons (13 months vs. 13.1 months, *p* = 0.2) [112]. A study by Labbè and colleagues in a cohort of 60 mutant *EGFR* patients found that *TP53* mutations were associated with worse PFS to first-line TKIs only when considering missense mutations (HR 1.91, CI 1.01–3.60, *p* = 0.04) [117]. These studies underline the importance of a categorization of *TP53* mutations; considering that, when taken together, *TP53* mutations were not able to reach significant associations with clinical outcome in advanced or early stage NSCLC, even in patients treated with third-generation TKIs [106,111,114,115,117,118,119,120]. The same observation was also highlighted in *EGFR*-mutated NSCLC patients by Jin et al., who found that *TP53* had the higher co-mutation rate with respect to other genic alterations (72.5%), but showed no associations with survival [121]. On the other hand, studies on Asian patients found that *TP53* mutations are able to predict PFS of *EGFR*-mutated patients treated with both first- or second- and third-generation *EGFR*-TKIs (HR: 2.02; 95% CI: 1.04–3.93, *p* = 0.038 and HR: 2.23, 95% CI 1.16–4.29, *p* = 0.017, respectively) [122]. The same results were achieved in a small case series of gefitinib-treated patients, where *TP53* mutations affected exclusively short- or intermediate responders (66.7% vs. 0, *p* = 0.009), and by a study by Yu and colleagues, who found that *TP53* mutations in pre-treated patients predicted shorter time-to-progression (HR 1.7, *p* = 0.006) [123,124]. An interesting study by Roeper and colleagues found that *TP53* mutations have a strong negative impact on the clinical outcome of EGFR-mutated patients (ORR, PFS and OS) whether considered as disruptive or non-disruptive; pathogenic or non-pathogenic; or exon 8 or non-exon 8 [125]. Another study found that *TP53* mutations are associated with early resistance to *EGFR*-TKIs, influencing the prognosis of short-responders to TKIs (<6 months, *TP53* mutations found in 87.5% of short-responders and in 16.7% of long-responders, *p* = 0.0002) [126]; these results were later confirmed by another study, which found that *TP53* mutations influences the prognosis of short-responders (<3 months, HR = 1.87, 95% CI 1.06–3.29, *p* = 0.03) and short-survivors (<6 months, HR = 2.73, 95% CI 1.20–6.21, *p* = 0.017) [127], and by another study, which found that 100% of non-responders to gefitinib were *TP53* mutated, with respect to 39% of responders (*p* < 0.001) [128]. It has recently been demonstrated that *TP53* mutations are independently associated with PFS in both first-, second-, and third-generation *EGFR*-TKIs (HR: 2.02; 95% CI: 1.04–3.93, *p* = 0.038 and HR: 2.23, 95% CI 1.16–4.29, *p* = 0.017, respectively) [122]. An interesting study by Tsui and colleagues explored the dynamics of resistance mechanisms to *EGFR*-TKI treatment through circulating DNA, finding that pretreatment of *TP53* mutations predicted a worse OS of patients (HR 0.43, 95% CI 0.2.0.97, *p* = 0.035), and that *TP53* mutation was observed in a patient experiencing progressive disease and without T790M [129]. Results from positive studies demonstrating a role of *TP53* mutations in predicting the clinical outcome of *EGFR*-mutated patients are resumed in Table 3.

It has been highlighted that the different methodologies used to detect *TP53* mutations have different sensitivities, as next-generation sequence technologies have led to an assessment of a greater rate of mutations in the case series; moreover, massive parallel sequencing has led to the sequencing of the entire *TP53* gene, highlighting mutations out of the DNA binding domain.

**Figure 1:**
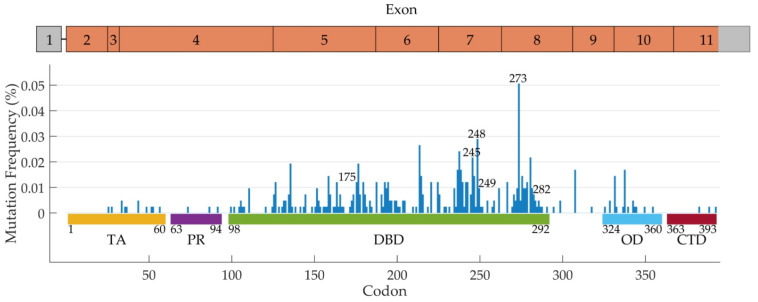
Frequency of *TP53* mutations in *EGFR*-mutated NSCLC patients, extracted by cBioPortal (https://www.cbioportal.org/, accessed on 28 January 2022). The percentage of *TP53* codon mutations along all gene exons, and the most frequently mutated codons are reported. TA: transactivation domain; PR: proline rich domain; DBD: DNA binding domain; OD: oligomerization domain; CTD: carboxy-terminal domain.

Two meta-analyses were also conducted, to better elucidate the role of *TP53* in mutant *EGFR* patients. The pooled results from 11 studies (1049 *EGFR*-mutated patients) highlighted that patients with concomitant *TP53* mutations have worse PFS (HR 1.76, 95% CI 1.44–2.16, *p* < 0.001) and OS (HR 1.83, 95% CI 1.47–2.29, *p* < 0.001). To better estimate the role of such mutations in predicting the response to EGR-TKIs, the authors performed a subgroup analysis of TKI-treated patients. The results remained significant both for patients receiving a TKI as a first-line therapy (HR for PFS 1.69, 95% CI 1.25–2.27, *p* = 0.001; HR for OS 1.94, 95% CI 1.36–2.76, *p* < 0.001) and for patients receiving TKIs in an all lines setting, for PFS and OS (HR for PFS 1.99, 95% CI 1.62–2.44, *p* = 0.001; HR for OS 1.93, 95% CI 1.45–2.58, *p* < 0.001) [130]. Another meta-analysis based on 2979 *EGFR*-mutated patients confirmed a worse OS for *TP53* mutated patients versus wt *TP53* patients (HR 1.73, 95% CI 1.22–2.44, *p* = 0.002). Interestingly, in a subgroup analysis, patients treated with TKIs had a worse PFS compared to other patients (HR 2.18, 95% CI 1.42–3.36, *p* < 0.001), even though this data was not confirmed by ORR analysis (RR 1.15, 95% CI 0.92–1.44, *p* = 0.212) [131]. Even though, in these studies, a categorization of *TP53* mutations was not performed, a clear role for *TP53* mutations as a negative factor for PFS and OS in *EGFR*-mutated patients treated with *EGFR*-TKIs was highlighted. In a study based on TCGA data, Wang et al., found that patients carrying *TP53* and *EGFR* co-mutations had a worse OS with respect to wt/wt patients (38.4 months vs. 51.9 months, *p*  =  0.023) [132]. Other indications highlight that *TP53* mutations are able to influence the response to TKIs depending on the type of *EGFR* alterations. Considering that patients with *EGFR* exon 19 deletions usually have a major benefit from first-line *EGFR*-TKIs compared to other *EGFR* mutations, it has been reported that *TP53* mutations are able to mainly affect the ORR and PFS of this subgroup of patients [114,133,134,135]. On the other hand, a recent study from the BENEFIT trial cohort investigated the *TP53* mutations in liquid biopsies of *EGFR*-mutated patients and found that exon 19 deletions and *TP53* co-mutations were predictors of better PFS and OS compared to exon 21 L858R/*TP53* co-mutations (HR for PFS 1.53, *p* = 0.02; HR for OS 0.77, *p* = 0.37). The same study also demonstrated a shorter PFS and OS for *TP53* mutated patients vs. *TP53* wt ones (HR for PFS 0.66, 95% CI 0.48–0.89, *p* = 0.007; HR for PFS 0.54, 95% CI 0.40–0.74, *p* < 0.001), with *TP53* mutations affecting the PFS and OS of patients with *EGFR* L858R mutations more than those with an exon 19 deletion. Interestingly, the authors also highlighted that patients with *TP53* exon 6 and 7 mutations experienced worse PFS and OS, with a role in predicting prognosis for exon 5 *TP53* mutations when categorized as disruptive and non-disruptive (OS HR = 2.04, 95% CI 0.99–4.19, *p* < 0.005) [25]. Recent results based on the CTONG 0901 trial identified that patients with *TP53* mutations in exons 4 or 7 experience worse PFS and OS with respect to patients with mutations affecting other exons of the gene or wild-type *TP53* (PFS 9.4, 11.0, and 14.5 months, respectively *p* = 0.009; OS 15.8, 20.0, and 26.1 months, respectively *p* = 0.004) [136]. The frequencies of *TP53* mutations in *EGFR*-mutated patients are reported in Figure 1.

### 5.2. TP53 Mutations in EGFR-Positive NSCLC: Phenotypic Changes

*TP53* mutations also play a pivotal role in the histologic transformation to small-cell lung cancer (SCLC), a mechanism of resistance to first- and second-generation *EGFR*-TKIs known since 2011 [62]. The inactivation of genes like RB1 and *TP53* play a crucial role in this transformation, as RB1 and *TP53* co-alterations present in patients’ tissues at baseline are more likely to predict histologic transformation, with respect to the presence of only one mutation, and the presence of both mutations is a significant prognostic factor [137,138,139]. In this setting, it seems that *TP53* mutation confers genomic instability to cancer cells resulting in a facilitated cell plasticity and phenotype reprogramming. The frequency of this evolution is about 3–5% and, usually, *EGFR* mutation is maintained [139,140,141].

Transformation to SCLC has also been observed as a mechanism of resistance to third-generation *EGFR*-TKIs used as a second-line treatment, with a frequency between 2 and 15%. This mechanism has been noticed with loss of T790M, but also when the resistance mutation is maintained, suggesting a focal and clonal tumor evolution [69,142]. When osimertinib is used as a first-line of treatment, transformation to SCLC has occurred in 4% of cases, as indicated by Schoenfeld and colleagues [54]. Transformation toward squamous cell carcinoma has been identified after treatment with osimertinib in II or later line and in I line, with a percentage of 9% and 7% respectively [54].

Recent results highlight that different *TP53* mutations influence, in diverse ways, the response to *EGFR*-TKIs in cell lines. In particular, some mutations are associated with primary resistance to *EGFR*-TKIs, while others can induce epithelial-mesenchymal transition (EMT) as an acquired resistance mechanism [141]. Considering that *TP53* participates in the regulation of the *EGFR* pathway [107], and that different mutations induce different mechanisms in different cell line models, it could be interesting to better investigate the cellular functions of the different *TP53* mutants. In this regard, a classification based on the disturbance grade of the p53 protein has been proposed [111], and several studies have confirmed that *TP53* mutations have different roles at a cellular level, and that some types of mutations are associated with oncogenic GOF [142]. Even though many studies of different case series brought interesting results based on this classification, which mutations and through which cellular mechanisms remain unanswered questions. Moreover, a study by Wei and colleagues highlights that *TP53* mutations are generally involved in resistance and primary and metastatic relapse, but different *TP53* exon alterations are involved in different mechanisms [143], and there is evidence that *TP53* mutations are found more frequently in association with *EGFR* mutations [144,145,146], suggesting that a subset of *EGFR*-NSCLC could have a “double-oncogene” addition, for the role that such mutations display.

## 6. Conclusions

NSCLC is the primary cause of cancer-related deaths. Several pieces of evidence suggest that *TP53* mutations are able to be used to identify a subset of patients with worse prognosis and a worse response to *EGFR*-TKIs; thus, identifying the different mutations associated with different outcomes. Given that *TP53* exerts its functions through a wide range of cellular pathways, it would be necessary not only to understand the function of a single mutation, but to consider how this mutation could affect which pathway, though which cellular and molecular mechanisms lie at the base of these processes still has to be elucidated. For this reason, the more that is revealed about the role of these gene mutations in NSCLC, the better we will understand the role in primary or acquired resistance to EGFR-TKIs in this malignancy. What we can assume so far is that, as observed in other tumors, *TP53* is a prognostic factor; however, after many reports concerning its effect on the efficacy of *EGFR*-TKIs, we now need to understand some of the molecular processes that link such mutations and resistance to *EGFR*-targeted therapy, as this could guide resistance to therapy. The link between *TP53* mutations and targeted therapy response should be considered a starting point for new investigations, and further studies are needed to investigate these mechanisms to effectively predict responsiveness and survival; thus, better tailoring targeted therapy for *EGFR*-NSCLC patients.

## Figures and Tables

**Table 1:** Phase III trials comparing *EGFR* inhibitors to chemotherapy in first-line treatment.

Study	Treatment Arms	mPFS (Months)	mOS (Months)	ORR (%)	Ref.
IPASS	Gefitinib vs. carboplatin/paclitaxel	9.8 vs. 6.4 *p* < 0.001	21.6 vs. 21.9 *p* = 0.99	71.2 vs. 47.3	[15]
NEJ002	Gefitinib vs. carboplatin/paclitaxel	10.8 vs. 5.4 *p* < 0.001	27.7 vs. 26.6 *p* = 0.48	73.7 vs. 30.7	[13]
WJT0G3405	Gefitinib vs. cisplatin/docetaxel	9.2 vs. 6.3 *p* < 0.0001	36.0 vs. 39.0	62.1 vs. 32.2	[16]
OPTIMAL	Erlotinib vs. carboplatin/gemcitabine	13.1 vs. 4.6 *p* < 0.0001	22.8 vs. 27.2 *p* = 0.27	83.0 vs. 36.0	[17]
EURTAC	Erlotinib vs. cisplatin/docetaxel	9.7 vs. 5.2 *p* < 0.0001	19.3 vs. 19.5 *p* = 0.87	64.0 vs. 18.0	[18]
ENSURE	Erlotinib vs. cisplatin/gemcitabine	11 vs. 5.6	26.3 vs. 25.5	62.7 vs. 33.6	[20]
CONVINCE	Icotinib vs. cisplatin/pemetrexed	11.2 vs. 7.9 *p* = 0.006	30.5 vs. 32.1 *p* = 0.89	NR	[21]
LUX-Lung 3	Afatinib vs. cisplatin/Pemetrexed	11.1 vs. 6.9 *p* = 0.001	28.2 vs. 28.2 *p* = 0.39	56.1 vs. 22.6	[22]
LUX-Lung 6	Afatinib vs. cisplatin/gemcitabine	11.0 vs. 5.6 *p* < 0.0001	23.1 vs. 23.5 *p* = 0.61	66.9 vs. 23.0	[23]
AURA 3	Osimertinib vs. platinum/pemetrexed	10.1 vs. 4.4 *p* < 0.001	NA	26.8 vs. 22.5	[24]
CTONG	Erlotinib vs. gefitinib	13.2 vs. 11.1	22.4 vs. 20.7	NR	[25]
LUX-Lung 7	Afatinib vs. gefitinib	13.7 vs. 11.5 *p* = 0.007	27.9 vs. 24.5	70 vs. 56	[26]
FLAURA	Osimertinib vs. erlotinib or gefitinib	18.9 vs. 10.2 *p* < 0.001	NR	80.0 vs. 76	[27]

mPFS: median Progression-Free Survival. mOS: median Overall Survival. ORR: Objective Response Rate. NR: Not Reported.

**Table 2:** Ongoing clinical trials in Osimertinib-resistant *EGFR*-mutated NSCLC.

Clinical Trial Number	Phase	Treatment Arms	Primary Endpoint	Link to the Clinical Trial
NCT03515837	III	Pembrolizumab + pemetrexed + chemo vs. placebo + pemetrexed + chemo	PFS, OS	https://clinicaltrials.gov/ct2/show/NCT03515837 (accessed on 28 January 2022)
NCT03778229	II	Osimertinib + savolitinib	ORR	https://www.clinicaltrials.gov/ct2/show/NCT03778229 (accessed on 28 January 2022)
NCT03944772	II	Osimertinib + savolitinib vs. osimertinib + gefitinib vs. osimertinib + necitumumab vs. durvalumab + carboplatin + pemetrexed	ORR	https://www.clinicaltrials.gov/ct2/show/NCT03944772 (accessed on 28 January 2022)
NCT03940703	II	Tepotinib + osimertinib	Safety, ORR	https://clinicaltrials.gov/ct2/show/NCT03940703 (accessed on 28 January 2022)
NCT04136535	II	Pemetrexed and carboplatin with or without anlotinib	PFS	https://www.clinicaltrials.gov/ct2/show/NCT4136535 (accessed on 28 January 2022)
NCT03532698	II	Osimertinib + aspirin	ORR	https://www.clinicaltrials.gov/ct2/show/NCT03532698 (accessed on 28 January 2022)
NCT04316351	II	Toripalimab + pemetrexed + anlotinib	ORR	https://www.clinicaltrials.gov/ct2/show/NCT04316351 (accessed on 28 January 2022)
NCT03784599	I/II	Trastuzumab emtansine and osimertinib	Safety, ORR	https://www.clinicaltrials.gov/ct2/show/NCT03784599 (accessed on 28 January 2022)
NCT03891615	I	Osimertinib + Niraparib	MTD	https://www.clinicaltrials.gov/ct2/show/NCT03891615 (accessed on 28 January 2022)
NCT03516214	I	Nazartinib and trametinib	MTD; RP2D	https://www.clinicaltrials.gov/ct2/show/NCT03516214 (accessed on 28 January 2022)

*EGFR*: Epidermal Growth Factor Receptor. NSCLC: Non-Small-Cell Lung Cancer. MDT: maximum tolerated dose. OS: Overall Survival. PFS: Progression-Free Survival. RP2D: recommended phase II dose. ORR: Objective response rate.

**Table 3:** Studies that found that *TP53* mutations are prognostic for *EGFR*-mutated NSCLC patients treated with *EGFR*-TKIs.

*TP53* Status	Number of Patients (Generation of TKI)	Result	Ref.
Non-disruptive mutations	193 (I)	OS	[105]
Any mutation	131 (I–II)	OS	[106]
Any mutation	116 (I–II)	OS	[107]
Exon 8 mutations	123 (I–II)	DCR, PFS, OS	[108]
Exon 8 mutations	136 (I–II)	OS	[109]
Exon 8 mutations	379 (I–II)	OS	[110]
Missense mutations	60 (I–II)	PFS	[111]
Any mutation	75; 82 (I–II; III)	PFS, PFS	[116]
Any mutation	18 (I)	TTP	[117]
Any mutation	374 (I–II)	TTP	[118]
Any mutation	28 (I)	TTP	[119]
Any mutation	132 (I)	PFS, OS	[121]
Any mutation	71 (I)	TTP, OS	[120]
Any mutation	50 (I)	OS	[122]
Any mutationExon 6, 7 mutations	368 (I)	PFS, OS	[129]
Exon 4, 7 mutations	256 (I)	PFS, OS	[130]

*EGFR*: Epidermal Growth Factor Receptor; TKI: Tyrosine Kinase Inhibitor; OS: Overall Survival; DCR: Disease Control Rate; PFS: Progression-Free Survival; TTP: Time-to-progression.

## Data Availability

The data presented in this study are available in the article.
